# Peripheral blood cell counts as correlates of insulin sensitivity in children and youth with type 1 diabetes: A retrospective study from western India

**DOI:** 10.1007/s12020-026-04721-5

**Published:** 2026-08-10

**Authors:** Sukeshini Khandagale, Shital Bhor, Chirantap Oza, Shruti Mondkar, Vinesh Kamble, Aditi Agnihotri, Anirudh Singh, Imaan Haque, Tejaswini Pachpor, Anuradha Khadilkar, Satyajeet Khare

**Affiliations:** 1 Symbiosis School of Biological Sciences (SSBS), Symbiosis International (Deemed University), Lavale, Pune, 412115 MH India; 2 Hirabai Cowasji Jehangir Medical Research Institute (HCJMRI), Pune, 411001 MH India; 3 Department of Biosciences and Technology, Dr. Vishwanath Karad MIT World Peace University, Pune, 411038 MH India

**Keywords:** Type 1 diabetes mellitus, insulin sensitivity, double diabetes, cellular correlates, peripheral blood

## Abstract

**Background:**

Type 1 diabetes mellitus (T1DM) is an insulin dependent diabetes treated with exogenous insulin therapy. A subset of individuals with T1DM develops reduced insulin sensitivity, a phenomenon often referred to as ‘double diabetes’, which is associated with an increased risk of complications. The pathophysiology of double diabetes in T1DM is not well understood. We hypothesize that changes in peripheral blood in T1DM may act as cellular correlates and provide insights into the development of double diabetes.

**Methodology:**

A retrospective study (*N* = 343) was performed at a tertiary care centre in western India. An estimated insulin sensitivity (eIS) score was calculated using the Coronary Artery Calcification in T1DM (CACTI) equation. A partial correlation, univariate, and multiple regression were performed to study the association of eIS with the components of hemogram (lymphocytes, neutrophils, monocytes, eosinophils, platelets, erythrocytes, and hemoglobin). Additionally, stratified multiple regression analyses were performed to assess the association of eIS with age, sex, and Body Mass Index (BMI).

**Results:**

Peripheral blood cell counts were associated with eIS in children and youth with T1DM. Across BMI, age, and sex strata, eosinophils consistently showed modest-to-moderate positive associations with eIS, whereas monocytes, neutrophils, lymphocytes, and platelets showed negative associations that varied by subgroup. In overall cohort, neutrophils and lymphocytes demonstrated statistically significant but minimal negative associations.

**Discussion:**

The development of double diabetes may occur independent of obesity and the cellular patterns observed paralleled those reported in non-obese populations. Larger studies are warranted to confirm these findings and to identify population-specific cellular correlates of declining insulin sensitivity in T1DM.

**Graphical abstract:**

**Figure:**
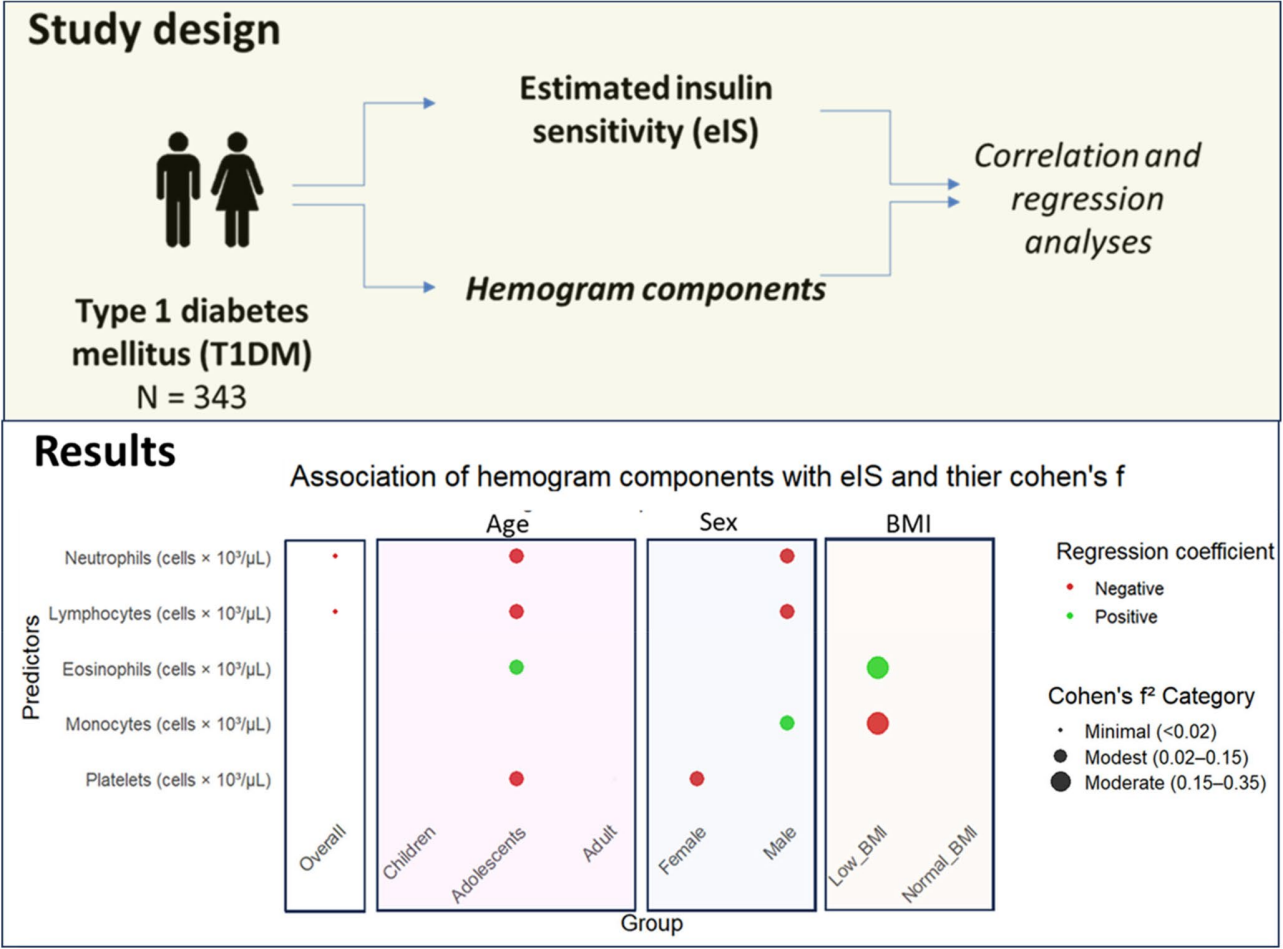

**Supplementary Information:**

The online version contains supplementary material available at 10.1007/s12020-026-04721-5.

## Highlights

Children and youth with type 1 diabetes exhibit normal-to-low BMI yet low estimated insulin sensitivity.

Peripheral blood cell counts associate with estimated insulin sensitivity in children and youth with type 1 diabetes.

Eosinophils associate positively, whereas neutrophils, lymphocytes, and platelets associate negatively with insulin sensitivity, with extent of association varying by age, sex, and BMI.

**Supplementary Information:**

The online version contains supplementary material available at 10.1007/s12020-026-04721-5.

## Introduction

Type 1 diabetes (T1DM) is a metabolic disorder characterized by insulinopenia requiring exogenous insulin therapy. Children with T1DM are at risk of developing complications associated with diabetes. The risk of complications increases with the decline in sensitivity to insulin, a phenomenon often referred to as “double diabetes” in individuals with T1DM [1]. However, there is no universally accepted diagnostic definition or standardized cut-off for double diabetes, and reported prevalence estimates vary depending on the criteria used [2]. Insulin sensitivity (IS) is estimated using a hyperinsulinemic euglycemic clamp (HEC) [3]. However, this method is invasive and expensive, thus, alternative surrogate methods have been developed that are routinely used to study molecular and cellular changes associated with IS [4]. In case of T1DM, the systemic insulin sensitivity is measured using indirect markers such as estimated insulin sensitivity (eIS) score [5].

In a non-T1DM context, the decline in insulin sensitivity (insulin resistance; IR) is estimated using indirect markers such as Homeostatic Model Assessment for Insulin Resistance (HOMA-IR) [6]. HOMA-IR has been associated with several hematological parameters including total white blood cells (WBCs), neutrophils, erythrocytes, neutrophil-to-lymphocyte ratio (NLR), and platelet-to-lymphocyte ratio (PLR) in overweight and obese children without diabetes [7–10]. In non-obese children, an association has been found with the platelet count [11]. IR has also been associated with total WBC count in young adults with T2DM [12]. Quantitative changes in the components of the hemogram have been found in children with new onset T1DM [4, 13]. However, studies on the association of the double diabetes with changes in cellular composition of peripheral blood are rare.

Eosinophils have been reported to associate positively with insulin sensitivity and inversely with insulin resistance [14]. In contrast, decreased monocyte counts and increased neutrophil counts have been associated with insulin resistance in adults with T1DM [15]. Platelets have also been implicated in insulin resistance and thromboinflammatory processes in T1DM [16]. However, whether these associations are present in children and young adults with T1DM remains unclear. Insulin resistance shows age and sex-based differences, being more prevalent in adolescent females [17]. Moreover, in T1DM insulin resistance can be found in children with low and normal BMI [18, 19]. Therefore, a stratified analysis that takes into account the effect of age, sex, and BMI for the association of IR with hemogram components is warranted.

To the best of our knowledge, data on hemogram correlates of insulin sensitivity in children with T1DM, adjusted for age, sex and BMI, are lacking. We hypothesize that systemic changes in the components of the hemogram are associated with insulin sensitivity in T1DM, and factors such as age, sex, and BMI influence these associations. These associations may provide cellular correlates to delineate the mechanisms underlying the decline in insulin sensitivity in T1DM and its subpopulation. We studied the association of the components of the hemogram with estimated insulin sensitivity in T1DM, and also based on age, sex, and BMI to explore whether distinct cellular correlates underlie differences across specific subgroups. Since there is no standard cut-off to diagnose IR or double diabetes using eIS, we used the eIS score as a continuous measure of insulin sensitivity.

## Methodology

### Study design

This is a retrospective observational study performed at a single tertiary care center in Western Maharashtra, India (Sweetlings cohort). For the retrospective analysis, children (5 to < 11yrs), adolescents (11 to < 18yrs), and young adults (18 to < 25yrs) diagnosed with T1DM were included [20]. The diagnosis of type 1 diabetes was based on clinical features e.g., hyperglycemia, clinical course consistent with insulin deficiency (low/absent C-peptide levels) and requirement of insulin therapy in accordance with the International Society for Pediatric and Adolescent Diabetes (ISPAD) Guidelines. Diagnosis was supported by assessment of autoantibodies and symptoms such as polyurea, polydipsia, and unexplained weight loss (ISPAD) [21]. Participants diagnosed with, or showing symptoms of complications associated with diabetes (nephropathy, dyslipidemia, retinopathy, etc.), and those on concomitant medication other than exogenous insulin therapy, were excluded. Pregnant women, lactating mothers were excluded. Participants with active infections, allergies, or any other autoimmune disorder, and participants with missing hematological, anthropometric, and clinical data were also excluded. Data on seven components of the hemogram (Neutrophils, Eosinophils, Lymphocytes, Monocytes, Erythrocytes, Hemoglobin, and Platelets) and four covariates [age, sex, BMI, and duration of diabetes (DoD)] was extracted.

### Data collection and estimation of insulin sensitivity

Blood samples were collected from participants in the fasting state (at least 8 h) during the comprehensive annual checks performed between 2022 and 2023. The data included complete blood count, lipid profile, HbA1c, insulin dosage, and anthropometric measurements such as height, weight, and waist circumference. Body Mass Index (BMI) was computed by dividing weight (kg) with square of height (m^2^). Duration of diabetes (DoD) was measured as period from the date of diagnosis till the date of sample collection. Specifications of the instruments utilized are provided (Supplementary Table 1). The Z scores for BMI were calculated according to Indian Academy of Pediatrics (IAP) guidelines for children of age 5yrs to 18yrs and for adults above 18yrs [22]. BMI values were standardized within the samples to derive the Z score. Estimated insulin sensitivity (eIS) was calculated using triglyceride concentration, insulin units per day per kg, diastolic blood pressure (DBP), and waist circumference as follows,

5 $$\begin{array}{lll}{\rm{eIS}} &=& {\rm{exp}} \, (4.1075-0.01299 \, [{\rm{waist, cm}}]\\&&-1.05819 \, [{\rm{insulin \, dose, \, daily \, units \, per \, kg}}] \\&&-\, 0.00354 \, [{\rm{triglycerides, \, mg/dL}}]\\&& - 0.00802 \, [{\rm{DBP, \, mm \, Hg}}]).\end{array}$$

### Correlation and regression analysis

The Shapiro-Wilk test was used to test the normality of the parameters in the data [23]. A simple spearman’s correlation analysis was performed between the calculated eIS and insulin dosage (IU/kg/day) to confirm its utility. We performed Spearman’s rank-based correlation on residuals of eIS and hemogram components, adjusting for age, sex, DoD, and BMIZ [24]. A residualized univariate regression was performed, adjusting for covariates to associate each hemogram component (predictor) to eIS (outcome). Multiple regression was performed [25], adjusting for covariates and confounders (age, sex, BMIZ, DoD, component of hemogram) to study independent association of each component of the hemogram with eIS. Both univariate and multiple regression models were tested for the significance of association (*p*-value < 0.05), and the direction and magnitude of the relationship by beta estimate. Effect sizes were estimated for significant (*p* < 0.05) variables [26]. The effects were considered as minimal (< 0.02), modest (0.02–0.15), moderate (0.15–0.35), and large (> 0.35).

### Stratified multiple regression analysis

 Multiple regression was performed within subgroups (only if *n* > 30) to test the association of components of hemogram with eIS based on age, sex, or BMI category. The subgroups for age were categorized as children, adolescents, and young adults. The subgroups based on BMI were categorized using Z scores as mentioned earlier for 5yrs to 18yrs, and WHO based Asian thresholds for adults [low BMI (BMI < 18.5 kg/m^2^), normal BMI (BMI: *≥*18.5 - <23 kg/m^2^), overweight and obese (BMI: *≥*23 kg/m^2^)]. To avoid over-adjustments, stratification variables were excluded from the corresponding regression model. Beta estimates, along with the *p*-value, are reported for each variable. Effect sizes were estimated for significant (*p* < 0.05) variables [26] .

## Results

### Cohort characteristics

**Fig. 1:**
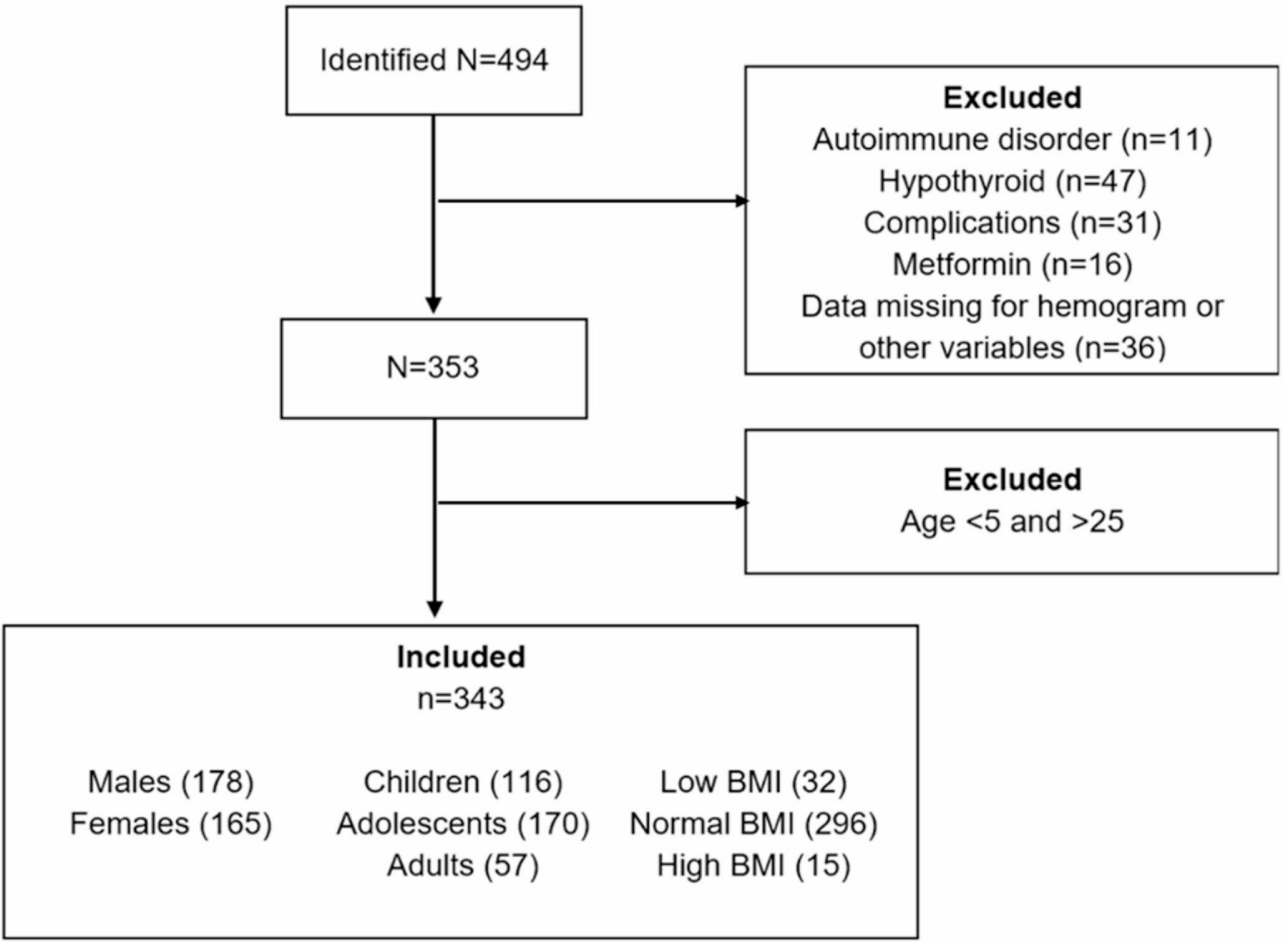
Consort flow diagram

Of the 494 records of participants with T1DM, those with hypothyroidism (*n* = 47), other autoimmune disorders (*n* = 11), complications (*n* = 31); those on metformin (*n* = 16), and those with missing records (*n* = 36) were excluded resulting in 353 eligible records. Further exclusion based on age resulted in retention of 343 records (Fig. 1). The median age of participants was 13 years [5–13, 15, 17–27] with a median diabetes duration of 5.2 years (10 days − 19.2 years). The cohort consisted of 116 (33.8%) children, 170 (49.6%) adolescents, and 57 (16.6%) young adults, 178 females and 165 males. Most participants had a normal BMI (children based on BMIZ, adults based on absolute BMI). In the cohort, low BMI (BMIZ <-2 or BMI < 18.5 kg/m²) were 32 (9.4%), normal BMI (BMIZ *≥*-2 to *≤ +* 1 or BMI *≥* 18.5 to < 23) were 296 (86.3%), and overweight and obese (BMIZ > + 1 or BMI *≥* 23) were 15 (4.3%).

Insulin sensitivity was estimated as eIS score. The utility of eIS was confirmed by its inverse correlation with the insulin dosage in units per kg per day (Supplementary Fig. 1). The median eIS was 3.9 (range: 1-10.4) and declined progressively from childhood through adolescence into young adulthood, but did not differ significantly between males and females or BMI groups (data not shown). Median values for all hemogram components were within expected reference ranges (Table 1).

**Table 1:** Demographic and Clinical details of the T1DM population

Parameter (unit)	Median (Min-Max)
Age (years)	13 (5–25)
DoD (years)	5.2 (10 days-19.2)
BMI (Kg/m²)	16.8 (11.6–27.5)
BMI Z score	-0.5 (-2.8–2.3)
HbA1C (%)	9.6 (5.9–18.7)
Waist circumference (cm)	65.2 (48–100)
Systolic blood pressure (mmHg)	103 (61–140)
Diastolic blood pressure (mmHg)	63 (40–169)
Hemoglobin (g/dL)	13.4 (9-17.7)
Cholesterol (mg/dL)	143 (75–252)
Triglyceride (mg/dL)	66 (26–241)
LDL (mg/dL)	85.6 (5.8–180)
HDL (mg/dL)	44 (23–115)
VLDL (mg/dL)	13.2 (5.2–48)
Neutrophils (cells × 10³/µL)	3.1 (0.3–8.4)
Eosinophils (cells × 10³/µL)	0.2 (0-1.1)
Lymphocytes (cells × 10³/µL)	2.2 (0.7-6)
Monocytes (cells × 10³/µL)	0.3 (0.1–0.7)
Platelets (cells × 10⁶/µL)	0.3 (0.1–0.7)
RBC (cells × 10⁶/µL)	4.9 (3.6–6.5)
NLR	1.4 (0.1–11)
MLR	0.1 (0-0.4)
PLR	144.4 (50–420)
Insulin units (IU/kg/day)	1.09 (0.2–2.4)
Estimated Insulin Sensitivity [eIS]	3.9 (1-10.4)

Note: *N* = 343

### eIS scores correlate with neutrophils, lymphocytes, and platelet counts

In unadjusted analysis by Spearman’s rank correlation (r_s_), HbA1c, triglycerides, VLDL, age, DoD, and BMIZ showed a significant negative correlation with eIS. eIS also correlated negatively with the hemogram components, viz. hemoglobin, neutrophil count, and NLR. Despite adjusting for age, sex, and BMIZ, the DoD showed a significant correlation with eIS (Table 2 ). Upon further adjusting for DoD, HbA_1C_ (r_s_=-0.2, *p* < 0.001) and triglycerides (r_s_=-0.3, *p* < 0.001) continued to show a negative association with eIS. Among hemogram components, neutrophils (r_s_=-0.1, *p* < 0.01), lymphocytes (r_s_=-0.1, *p* < 0.05), and platelets (r_s_=-0.12, *p* < 0.05) showed a low magnitude but significant negative correlation.

**Table 2:** Spearman’s correlation between estimated insulin sensitivity and differential hemogram components, and their estimated ratios

Variable	Unadjusted	Adj (Age + BMIZ + sex)	Adj (BMIZ + Age + DoD + sex)
Age (years)	-0.3***	0.03	0.02
Sex	0.1	0	-0.003
DOD (years)	-0.4***	-0.2***	0.1
BMI (Z score)	-0.1*	0.001	0.02
HbA1c (%)	-0.2***	-0.2***	-0.2***
Hemoglobin (g/dL)	-0.1*	-0.04	-0.1
Cholesterol (mg/dL)	-0.1	-0.04	-0.03
Triglyceride (mg/dL)	-0.4***	-0.3***	-0.3***
LDL (mg/dL)	-0.02	0.04	0.04
HDL (mg/dL)	0.05	0.01	-0.001
VLDL (mg/dL)	-0.4***	-0.3***	-0.3***
Neutrophils (cells × 10³/µL)	-0.2***	-0.2***	-0.1**
Eosinophils (cells × 10³/µL)	-0.02	-0.1	-0.04
Lymphocytes (cells × 10³/µL)	0.004	-0.1	-0.1*
Monocytes (cells × 10³/µL)	-0.1	-0.1	-0.1
Platelets (cells × 10⁶/µL)	-0.09	-0.10*	-0.12*
RBC (cells × 10⁶/µL)	-0.038	-0.04	-0.06
Neutrophil-Lymphocyte Ratio (NLR)	-0.2***	-0.1	-0.04
Monocyte-Lymphocyte Ratio (MLR)	-0.1	0.003	0.04
Platelet-Lymphocyte Ratio (PLR)	-0.1	-0.01	0.01

Note: DoD: Duration of Disease. *p* ≤ 0.05(*), pval ≤ 0.01(**), pval ≤ 0.001(***)

### Neutrophils, lymphocytes, and platelet count predict eIS

Univariate regression models, each adjusted for age, sex, BMIZ, and DoD, showed that neutrophils (B = -0.2, 95% CI: -0.3 to -0.03, *p* < 0.05), lymphocytes (B = -0.3, 95% CI: -0.5 to -0.1, *p* < 0.01), and platelets (B = -2.52, 95% CI: -4.5 to -0.5, *p* < 0.05) were significant negative predictors of eIS. In multiple regression analysis, neutrophils (B = -0.2, 95% CI: -0.3 to -0.01, *p* < 0.05) and lymphocytes (B = -0.3, 95% CI: -0.5 to 0.0, *p* < 0.05) were independent negative predictors of eIS whereas the platelet association was attenuated and no longer significant (Table 3). Effect sizes (f²) for associations in multiple regression were minimal (< 0.02) (Supplementary Table 2).

**Table 3:** Regression analysis of the association of eIS with hemogram components

Variable	B (estimate)	95% CI	*P*-value	Significance
Univariate regression analysis (*n* = 343)				
Neutrophils (cells × 10³/µL)	-0.2	[-0.3, -0.03]	0.01	*
Lymphocytes (cells × 10³/µL)	-0.3	[-0.5, -0.1]	0.01	*
Eosinophils (cells × 10³/µL)	0.2	[-0.7, 1]	0.7	
Monocytes (cells × 10³/µL)	-1.0	[-2.3, 0.5]	0.2	
Platelets (cells × 10⁶/µL)	-2.52	[-4.5, -0.5]	0.01	*
RBC (cells × 10⁶/µL)	-0.23	[-0.6, 0.15]	0.2	
Hemoglobin (g/dL)	-0.1	[-0.2, 0.1]	0.4	
**Multiple regression analysis** **(n = 343)**				
(Intercept)	7.8	[5.5, 10.1]	< 0.001	***
Neutrophils (cells × 10³/µL)	-0.2	[-0.3, 0]	0.03	*
Lymphocytes (cells × 10³/µL)	-0.3	[-0.5, 0]	0.02	*
Eosinophils (cells × 10³/µL)	0.5	[-0.4, 1.3]	0.3	
Monocytes (cells × 10³/µL)	0.6	[-1.1, 2.3]	0.5	
Platelets (cells × 10⁶/µL)	-1.61	[-3.7, 0.55]	0.1	
RBC (cells × 10⁶/µL)	-0.11	[-0.51, 0.28]	0.6	
Hemoglobin (g/dL)	0	[-0.2, 0.1]	0.7	
Age (years)	-0.1	[-0.1, 0]	< 0.001	***
BMI (Z score)	-0.1	[-0.3, 0.1]	0.4	
DoD (years)	-0.1	[-0.2, -0.1]	< 0.001	***
Sex Male	0.2	[-0.1, 0.6]	0.2	

Note: *p*val ≤ 0.05(*), *p*val ≤ 0.01(**), *p*val ≤ 0.001(***). Unstandardized regression coefficient (B)

### Neutrophils, Lymphocytes, eosinophils, and platelets are correlates for eIS in adolescents

Stratified multiple regression analyses conducted in children, adolescents, and young adults showed a differential association of eIS with hemogram components. Specifically, in adolescents, eIS showed a significant negative association with neutrophils (B= -0.2, 95% CI: -0.4 to -0.1, *p* < 0.05), lymphocytes (B = -0.4, 95% CI: -0.8 to -0.1, *p* < 0.05), and platelets (B = -3.07, 95% CI: -6.16 to 0.01, *p* < 0.05), whereas a positive association was observed with eosinophils (B = 2.2, 95% CI: 0.8 to 3.5, *p* < 0.01) (Table 4). Effect sizes (f²) for these associations were in the modest range (0.02–0.15) (Supplementary Table 2). No association of eIS with hemogram components was observed in children and young adults.

**Table 4:** Multiple regression in children, adolescents, and young adults adjusted for sex, BMI and DoD

Variable	B (estimate)	95% CI	*P* value	Significance
Multiple regression in children (*n* = 116)				
(Intercept)	2.5	[-2.7, 7.6]	0.3	
Neutrophils (cells × 10³/µL)	-0.2	[-0.4, 0.04]	0.1	
Lymphocytes (cells × 10³/µL)	-0.2	[-0.6, 0.1]	0.2	
Eosinophils (cells × 10³/µL)	0.3	[-1, 1.7]	0.6	
Monocytes (cells × 10³/µL)	-1	[-4.3, 2.3]	0.5	
Platelets (cells × 10⁶/µL)	0.33	[-3.09, 3.76]	0.9	
RBC (cells × 10⁶/µL)	0.15	[-0.8, 1.12]	0.7	
Hemoglobin (g/dL)	0.3	[-0.1, 0.6]	0.1	
DOD (years)	-0.2	[-0.4, -0.1]	< 0.001	***
BMI (Z score)	-0.2	[-0.6, 0.1]	0.1	
Sex Male	0.3	[-0.3, 0.8]	0.3	
**Multiple regression in adolescents** (*n = 170)*				
(Intercept)	8.9	[5.9, 11.9]	< 0.001	***
Neutrophils (cells × 10³/µL)	-0.2	[-0.4, -0.05]	0.01	*
Lymphocytes (cells × 10³/µL)	-0.4	[-0.75, -0.09]	0.01	*
Eosinophils (cells × 10³/µL)	2.2	[0.78, 3.52]	< 0.01	**
Monocytes (cells × 10³/µL)	1.2	[-1.05, 3.63]	0.3	
Platelets (cells × 10⁶/µL)	-3.07	[-6.16, 0.01]	0.04	*
RBC (cells × 10⁶/µL)	-0.30	[-0.8, 0.19]	0.2	
Hemoglobin (g/dL)	-0.1	[-0.24, 0.08]	0.3	
DOD (years)	-0.1	[-0.18, -0.07]	< 0.001	***
BMI (Z score)	-0.01	[-0.27, 0.26]	0.9	
Sex Male	0.1	[-0.39, 0.54]	0.7	
**Multiple regression in young adults** (*n* = 57)				
(Intercept)	9.1	[2.5, 15.8]	< 0.01	**
Neutrophils (cells × 10³/µL)	0.1	[-0.3, 0.4]	0.7	
Lymphocytes (cells × 10³/µL)	0.1	[-0.6, 0.8]	0.8	
Eosinophils (cells × 10³/µL)	-0.6	[-3.4, 2.1]	0.6	
Monocytes (cells × 10³/µL)	-0.2	[-4.7, 4.6]	1	
Platelets (cells × 10⁶/µL)	-4.6	[-11.5, 2.2]	0.2	
RBC (cells × 10⁶/µL)	-0.4	[-1.3, 0.5]	0.4	
Hemoglobin (g/dL)	-0.2	[-0.6, 0.2]	0.4	
DOD (years)	0	[-0.2, 0.1]	0.5	
BMI (Z score)	-0.1	[-0.6, 0.3]	0.6	
Sex Male	0.9	[-0.5, 2.3]	0.2	

### Neutrophils and lymphocytes in males, and platelets in females, are correlates for lower eIS

eIS remained significantly associated with neutrophils (B = -0.3, 95% CI: -0.5 to -0.1, *p* < 0.01) and lymphocytes (B = -0.4, 95% CI: -0.8 to -0.1, *p* = 0.05) in males. Interestingly, a positive association of monocytes (B = 2.9, 95% CI: 0.2 to 5.5, *p* < 0.05) was also observed among males. In females, only platelets showed a significant negative association with eIS (B = -3.96, 95% CI: -7.17 to -0.74, *p* < 0.05) (Table 5). Effect sizes (f²) for these associations were in the modest range (0.02–0.15) (Supplementary Table 2).

**Table 5:** Multiple Regression for Male and Female adjusting for age, duration of diabetes, and BMI

Variable	B (estimate)	95% CI	*P* value	Significance
Regression analysis in females (*n* = 165)				
(Intercept)	10.2	[6.5, 13.9]	< 0.001	***
Neutrophils (cells × 10³/µL)	0	[-0.2, 0.2]	0.8	
Lymphocytes (cells × 10³/µL)	-0.1	[-0.4, 0.2]	0.4	
Eosinophils (cells × 10³/µL)	0	[-1.2, 1.2]	0.9	
Monocytes (cells × 10³/µL)	-1	[-3.3, 1.3]	0.3	
Platelets (cells × 10⁶/µL)	-3.96	[-7.17, -0.74]	< 0.05	*
RBC (cells × 10⁶/µL)	-0.45	[-0.99, 0.08]	0.1	
Hemoglobin (g/dL)	0	[-0.2, 0.2]	0.8	
Age (years)	-0.1	[-0.1, 0]	< 0.05	*
DoD (years)	-0.1	[-0.2, -0.1]	< 0.001	***
BMI (Z score)	0	[-0.2, 0.3]	0.8	
**Regression analysis in males** (*n* = 178)				
(Intercept)	4.6	[1.3, 7.8]	< 0.01	**
Neutrophils (cells × 10³/µL)	-0.3	[-0.5, -0.1]	< 0.01	**
Lymphocytes (cells × 10³/µL)	-0.4	[-0.8, -0.1]	< 0.05	*
Eosinophils (cells × 10³/µL)	1.1	[-0.2, 2.4]	0.1	
Monocytes (cells × 10³/µL)	2.9	[0.2, 5.5]	< 0.05	*
Platelets (cells × 10⁶/µL)	0.8	[-2.1, 3.7]	0.6	
RBC (cells × 10⁶/µL)	0.5	[-0.1, 1.2]	0.1	
Hemoglobin (g/dL)	-0.1	[-0.3, 0.2]	0.7	
Age (years)	-0.1	[-0.2, 0]	< 0.05	*
DoD (years)	-0.1	[-0.2, 0]	< 0.01	**
BMI (Z score)	-0.3	[-0.5, 0]	< 0.05	*

Note: *p*val ≤ 0.05(*), *p*val ≤ 0.01(**), *p*val ≤ 0.001(***)

### Monocytes and eosinophils are associated with eIS in participants with low BMI

Stratification by BMI was feasible only for groups with low and normal BMI, as overweight/obese participant numbers were insufficient for analysis. In participants with normal BMI, no hemogram component was associated with eIS. On the contrary, the participants with low BMI showed a positive association with eosinophils (B = 3.3 95% CI: 0.4 to 6.2, *p* < 0.05) and a negative association with monocytes (B = -9.8, 95% CI: -17.4 to -2.2, *p* < 0.05) (Table 6). Effect sizes (f²) for these associations were in the moderate range (0.15–0.35) (Supplementary Table 2).

**Table 6:** Multiple Regression for participants with low BMI and normal BMI adjusting for age, sex and duration of diabetes

Variable	B (estimate)	95% CI	*P* value	Significance
Multiple regression in low BMI (*n* = 32)				
(Intercept)	5.2	[-1.4, 11.8]	0.1	
Neutrophils (cells × 10³/µL)	-0.1	[-0.6, 0.4]	0.8	
Lymphocytes (cells × 10³/µL)	0.1	[-0.8, 0.9]	1	
Eosinophils (cells × 10³/µL)	3.3	[0.4, 6.2]	< 0.05	*
Monocytes (cells × 10³/µL)	-9.8	[-17.4, --2.2]	< 0.05	*
Platelets (cells × 10⁶/µL)	3.8	[-8, 15.6]	0.5	
RBC (cells × 10⁶/µL)	-0.6	[1.6, 0.4]	0.3	
Hemoglobin (g/dL)	0.2	[-0.2, 0.6]	0.5	
Age (years)	0	[-0.2, 0.1]	0.7	
DOD (years)	0	[-0.1, 0.2]	0.9	
Male sex	0.7	[-0.8, 2.3]	0.3	
**Multiple regression in normal BMI ** (*n* = 296)				
(Intercept)	8.1	[5.6, 10.6]	< 0.001	***
Neutrophils (cells × 10³/µL)	-0.2	[-0.3, 0.006]	0.1	
Lymphocytes (cells × 10³/µL)	-0.2	[-0.4, 0.1]	0.2	
Eosinophils (cells × 10³/µL)	0.1	[-0.9, 1.1]	0.9	
Monocytes (cells × 10³/µL)	0.8	[-1.03, 2.7]	0.4	
Platelets (cells × 10⁶/µL)	-1.83	[-4.12, 0.4]	0.1	
RBC (cells × 10⁶/µL)	-0.12	[-0.56, 0.32]	0.6	
Hemoglobin (g/dL)	-0.03	[-0.2, 0.1]	0.6	
Age (years)	-0.09	[-0.1, -0.04]	< 0.001	***
DOD (years)	-0.1	[-0.2, -0.1]	< 0.001	***
Male sex	0.2	[-0.1, 0.6]	0.2	

## Discussion

In this retrospective study in children and youth with T1DM, we found that insulin sensitivity (IS) is associated with multiple components of the hemogram. Neutrophils, lymphocytes, and platelets showed a significant negative correlation with eIS, whereas NLR, MLR, and PLR did not correlate. Regression analyses confirmed that neutrophils and lymphocytes were independent predictors of eIS, although with minimal effect sizes. The stratified analysis revealed a small but significant inverse association of eIS with neutrophils and lymphocytes and a positive association with eosinophils in adolescents. This association was not found in children and adults. eIS was associated with neutrophils, lymphocytes, and monocytes in males; and platelets in females. The participants with low BMI showed a moderate association of eIS with eosinophils and monocytes. Overall, eosinophils associated positively with the eIS whereas neutrophils, lymphocytes, and platelets showed a negative association. Our results suggest that the components of hemogram associate with eIS in children and youth with T1DM and that these association are influenced by age, sex, and BMI.

In our cohort, the median HbA1c suggested suboptimal glycemic control while lipid profile and hemogram values were largely within normal ranges. Most participants showed NLR, MLR and PLR within the normal range suggesting absence of active inflammation. Most participants had normal BMI, yet low eIS (< 3.5) was observed in 144 individuals suggesting that the development of insulin resistance in T1DM may not be associated with obesity. The results also suggest that, Indian children with T1DM may have a higher prevalence of IR compared to global trends. eIS declined with increasing age but did not show a significant difference between sexes.

The inverse association of triglycerides and HbA1c with eIS is consistent with established metabolic markers of insulin sensitivity. Among hemogram components, neutrophils showed a small but significant negative association with eIS. Neutrophils have been positively associated with IR in T2DM and negatively with IS in adults with T1DM [15]. Higher neutrophil count has been associated with an increase in circulating elastase production. The elastase has been shown to degrade the insulin receptor GLUT3, resulting in the decline of insulin sensitivity in the mouse model of T2DM [27, 28]. However, these studies associate neutrophils to insulin resistance in presence of obesity which is not observed in our cohort. In our T1D cohort, reduced insulin sensitivity may instead relate to chronic hyperglycaemia, glycaemic variability, oxidative stress, and low-grade systemic inflammation, which can activate circulating neutrophils. Higher proportions of neutrophils have been associated with the development of complications in T1DM through elastase [29, 30]. Thus, the negative association between neutrophils and eIS in our cohort may reflect early molecular mechanisms that predispose to complications. These findings should be considered as associative and hypothesis generating. In stratified analysis, males showed neutrophils as a significant predictor of eIS with modest effect size, but not females, consistent with known sex-related differences in neutrophil biology. Notably, neutrophils predicted eIS in adolescents to similar effect size, but not in children and adults. The age-specific variation of neutrophil with insulin sensitivity has not been reported in T1DM and could be due to differences in insulin sensitivity levels among other factors.

In our analysis, we also found lymphocytes to be negatively associated with eIS. The association was small but significant. Lymphocytes have been associated with reduced glucose tolerance in non-obese adults with T2DM [31]. Consistent with these observations, participants in our cohort were predominantly non-obese and therefore may exhibit a similar pattern of association. In our stratified analysis, lymphocytes could independently predict eIS in males, but not females, and only in adolescents. However, the functional association between the two requires further investigation.

The correlation and univariate regression analyses suggested an association of platelets with eIS. However, this association was not retained in multiple regression analysis, suggesting confounding by other components of the hemogram. In stratified analyses, platelets were negatively associated with eIS in adolescents, consistent with their positive association with insulin resistance reported in children and adolescents without diabetes [11, 32], highlighting the influence of age and BMI on this association. Insulin resistance has been associated with oxidative stress resulting in reduced lifespan of platelets. Omental adipose tissue has been linked to secretion of adipokines including thrombopoietin which increases the platelet count in response to platelet cell death [33, 34]. This modulation of platelet activity has been demonstrated in adults with T1DM [16]. In our cohort, platelets were also associated negatively with eIS in females but not males, consistent with the findings in adolescent girls [35]. The mechanistic role of platelets in the decline of insulin sensitivity specifically in females is not completely understood.

Other cell types did not show an association with eIS in regression analysis; however, in stratified analysis, eosinophils were positively associated with eIS in participants with low BMI. This finding aligns with a prior report showing higher eosinophil counts associated with lower insulin resistance in adults with risk of T2DM [14]. Eosinophils have been implicated in metabolic regulation through anti-inflammatory pathways and may support insulin sensitivity. In contrast to neutrophils, they may represent a counter-regulatory immune component. However, in our study, eosinophil counts were not assessed mechanistically, and their association with eIS should be considered exploratory rather than causal. Monocytes were negatively associated with eIS in participants with low BMI, whereas a positive association was observed specifically in males. These divergent associations have not been previously reported in T1DM subgroups and require further investigation. Due to small sample size in low BMI group the result should be considered an exploratory finding.

### Strengths and limitations

This is the first study to investigate the association of hemogram components with insulin sensitivity in children with T1DM. We excluded participants on oral hypoglycemic agents, ensuring that results are not confounded by the treatment. However, several limitations should be noted. The CACTI-derived equation was originally developed in predominantly non-Hispanic white adults with type 1 diabetes and, although explored in adolescents, its validation in younger (prepubertal) and ethnically diverse populations remains limited. Due to the cross-sectional, retrospective nature of the study, we could not assess the causal associations between the lower eIS and changes in components of the hemogram. Considering the transient nature of hematological measurements, the functional association needs investigation in a separate cohort. Moreover, covariates such as socio-economic status, physical activity, hemostatic episodes, and pubertal status with respect to tanner stages were also not directly included in the analysis. The results for the subgroup analysis in young adults and low BMI participants should be interpreted as exploratory findings. The study lacks healthy or obese non-diabetic control groups.

In conclusion, neutrophils and lymphocytes were identified as independent correlates of estimated insulin sensitivity in children and youth with T1DM, consistent with findings from non-obese populations without diabetes. Adolescents showed robust associations of eIS with neutrophils and lymphocytes; however, the underlying factors, including possible developmental influences, remain unclear. Sex-specific patterns were also evident, with neutrophils and platelets associating with eIS in males and females respectively. Despite the modest to moderate effect sizes, the associations suggest a role of immune cells in the decline in insulin sensitivity. These correlates should be validated in larger cohorts to test their application as screening tools in clinical settings. Future work should explore mechanistic and age-specific pathways, and evaluate models that can better capture immune-metabolic interactions across developmental stages.

## Supplementary Information

Below is the link to the electronic supplementary material.

Supplementary Material 1

## Data Availability

https://github.com/macdlab/2025_SK_T1DM_MR.
