# Supplementary material for: Peripheral blood cell counts as correlates of insulin sensitivity in children and youth with type 1 diabetes: A retrospective study from western India
Source: Endocrine. 2026 Aug 10;91(1):269. doi: 10.1007/s12020-026-04721-5 (PMC13457249; doi:10.1007/s12020-026-04721-5)
Supplement: Supplementary file 1 [file 12020_2026_4721_MOESM1_ESM.docx]

Supplementary information

**Peripheral blood cell counts as correlates of insulin sensitivity in children and youth with Type 1 Diabetes: A retrospective study from Western India**

Sukeshini Khandagale1, Shital Bhor2, Chirantap Oza2, Shruti Mondkar2, Vinesh Kamble1, Aditi Agnihotri1, Anirudh Singh1, Imaan Haque1, Tejaswini Pachpor3, Anuradha Khadilkar2, Satyajeet Khare1

1. Symbiosis School of Biological Sciences (SSBS), Symbiosis International (Deemed University), Lavale, Pune, MH 412115, India

2. Hirabai Cowasji Jehangir Medical Research Institute (HCJMRI), Pune, MH 411001, India

3. Dr. Vishwanath Karad MIT World Peace University

Correspondence: [sukeshinik5@gmail.com](mailto:sukeshinik5@gmail.com), [anuradhavkhadilkar@gmail.com](mailto:anuradhavkhadilkar@gmail.com), [satyajeetkhare@gmail.com](mailto:satyajeetkhare@gmail.com)

**Supplementary table 1: Information of the instruments employed for the blood and physical analysis**

| **Analyte** | **Instrument details** |
| --- | --- |
| CBC | Horiba yumizen H500 |
| Lipid profile | Selectra Pro S (Fully automated) |
| HbA_1C_ | HPLC BioRad D10, Germany (Fully automated) |
| Weight | Bioelectrical Impedance Analyzer (TANITA analyzer BC-420) |
| Height | Seca portable stadiometers up to 0.1 cm accuracy |

**Supplementary Table 2: Post-hoc test for f^2^ for each multiple regression (MR) model**

| Method | Sample Size (n) | u (no of predictors) | v (residual degrees of freedom) | Adj R2 (Squared Multiple Correlation) | f2 (Cohen’s effect size) | Significance level (α) |
| --- | --- | --- | --- | --- | --- | --- |
| Overall MR | 343 | 11 | 331 | 0.2 | 0.2 | 0.05 |
| Children MR | 116 | 10 | 105 | 0.2 | 0.2 | 0.05 |
| Adolescents MR | 170 | 10 | 159 | 0.2 | 0.2 | 0.05 |
| *Adults MR | 57 | 10 | 46 | -0.06 | 0.1 | 0.05 |
| Male MR | 165 | 10 | 154 | 0.2 | 0.3 | 0.05 |
| Female MR | 178 | 10 | 167 | 0.2 | 0.2 | 0.05 |
| Low BMI MR | 32 | 10 | 21 | 0.3 | 0.5 | 0.05 |
| Normal BMI MR | 296 | 10 | 285 | 0.2 | 0.3 | 0.05 |

**Note**: The data for high BMI is low (n=15) therefore, regression could not be performed

*Power calculation performed using R^2^ (0.12), not adjusted R2 which was negative.

**Supplementary Figure 1:** Spearman’s correlation of insulin units per kg per day with insulin sensitivity measured by eIS score (n=343).
